# Relative Stability of Boron Planar Clusters in Diatomic Molecular Model

**DOI:** 10.3390/molecules27051469

**Published:** 2022-02-22

**Authors:** Levan Chkhartishvili

**Affiliations:** 1 Department of Engineering Physics, Georgian Technical University, 77 Merab Kostava Avenue, Tbilisi 0160, Georgia; levanchkhartishvili@gtu.ge; 2 Boron and Powder Composite Materials Laboratory, Ferdinand Tavadze Metallurgy and Materials Science Institute, 8b Elizbar Mindeli Street, Tbilisi 0186, Georgia

**Keywords:** planar cluster, charge state, bond length, specific binding energy, relative stability, formation probability, boron

## Abstract

In the recently introduced phenomenological diatomic molecular model imagining the clusters as certain constructions of pair interatomic chemical bonds, there are estimated specific (per atom) binding energies of small all-boron planar clusters B*_n_*, *n* = 1–15, in neutral single-anionic and single-cationic charge states. The theoretically obtained hierarchy of their relative stability/formation probability correlates not only with results of previous calculations, but also with available experimental mass-spectra of boron planar clusters generated in process of evaporation/ablation of boron-rich materials. Some overestimation in binding energies that are characteristic of the diatomic approach could be related to differences in approximations made during previous calculations, as well as measurement errors of these energies. According to the diatomic molecular model, equilibrium binding energies per B atom and B–B bond lengths are expected within ranges 0.37–6.26 eV and 1.58–1.65 Å, respectively.

## 1. Introduction

Nanoboron and boron-rich nanomaterials are of current academic and practical interests because of their widely variable interatomic bonding mechanism and related unique complex of physical–chemical properties useful in technological applications—see some of the reviews in the last decade [1,2,3,4,5,6,7,8,9].

Among them, the all-boron clusters B*_n_*, *n* = 2, 3, 4, …, as individual species in the gas phase, play an important role, as they can serve for building blocks in the boron-rich solids chemistry [10]. For example, a quasi-planar boron cluster B_35_ with a double-hexagonal hole at the center has been reported [11] as a flexible structural motif for borophene allotropies, as it can be used to construct atom-thin boron sheets with various hexagonal hole densities.

In this regard, it should be noted that, depending on the number of atoms and also formation kinetics, boron clusters can take several different shapes. Joint experimental studies and computational simulations revealed [12] that boron clusters, which favor (quasi)planar, i.e., 2D, structures up to 18 atoms, prefer 3D structures beginning at 20 atoms. The B_20_ neutral cluster was found to have a double-ring tubular ground structure. As for the B_20_^−^ anion, its tubular structure was shown to be almost isoenergetic to 2D structures. Thus, the usually observed 2D-to-3D structural transition suggests that B_20_ may be considered as the embryo of thinnest single-walled boron nanotubes. According to the QC (Quantum Chemical) and DFT (Density Functional Theory) investigations [13], there are two structural transitions that are expected in boron clusters: the second transition from double-ring system into triple-ring one occurs between B_52_ and B_54_.

At low (namely, from 7 to 20) nuclearities, i.e., for (quasi)planar boron clusters, the separate quantum rules of in- and out-of-plane bonding were obtained [14], using the free-particle-on-disk and rectangle models combined with DFT electronic structure calculations.

In this Introduction, the quite-rich data available on boron clusters synthesis methods, binding parameters and their potential applications are only briefly discussed.

### 1.1. Synthesizing

Boron quasi-planar clusters can be formed in process of thermal vaporization [15,16,17,18,19,20,21], bombardment with high-energy particles [22], even grinding of boron-rich solid materials [23,24,25] and mainly by their laser ablation [26,27,28,29].

An effective method of thermal generating of pure boron cluster-ions for their further use as a plasma-process feed gas was proposed by Becker [18]. Chamber’s electrode material is a compound of boron with Me metal(s) thermally decomposable within a suitable temperature range to provide boron in the vapor, but other species are substantially not in the vapor states. Magnetic confinement of the simultaneously released electrons causes numerous collisions, resulting in boron vapor ionization to the plasma state. This plasma is then extracted and accelerated at a suitable energy toward the workpiece. Created in this way, boron clusters can be self-assembled into nanostructures [19].

As early as in References [23,24], electron microscopic study of the elementary boron powder structure revealed that ultrafine particles (of ≤200 Å size) of freely grown boron are characterized by a stable 2D shape with almost hexagonal profile and aspect ratio of ~20:1. Computer simulations were performed [25] to model structural relaxation in 2D-clusters mimicking these boron small particles.

Theoretically, using DFT, a growth path for small boron clusters B*_n_* was discussed [30] with a size range and isomers structure. The thermochemical parameters that were determined by using coupled-cluster theory calculations suggested [31] the evolution of geometry and resonance energy of B*_n_* clusters through the number of B–B bonds. Based on the known geometrical characteristics of boron clusters, their general growth mechanism was proposed in Reference [32]. Moreover, a systematic structural investigation of B*_n_* clusters established a picture of their growth behavior [33].

### 1.2. Structure and Binding

According to References [15,34], the diboron molecule B_2_ experimental dissociation energy is within range of 2.82 ± 0.24 or 2.69 ± 0.43 eV, respectively. The absorption transition at 3200–3300 Å observed in B_2_ indicated [35] that its ground electronic state should be the lower state of Σ-type. Moreover, as B_2_ was not observed via ESR (Electron Spin Resonance), the ground electronic state was identified with ^3^Σ_g_^−^. An accurate CI (Configuration Interaction) calculation confirmed [36] that mentioned transition is from the first excited state of ^3^Σ_u_^−^ type. Molecular binding energy for B_2_ at the HF (Hartree–Fock) level of theory [37] is 2.861 eV, and its scaled ground-state harmonic frequency equals to 0.120 eV. From the DFT calculations [30], these parameters are 2.718 and 0.134 eV, respectively. By using the MO (Molecular Orbitals) method, the dissociation energy of B_2_ ground state was calculated to be 2.71 eV [38]. Handbook [39] recommends the diboron molecule ground electron state dissociation energy of 3.02 eV. In this state, its bond length and vibration quantum are 1.590 Å and 0.130 eV, and in the first excited state, they are 1.625 Å and 0.116 eV, respectively. The term of corresponding transition equals to 3.79 eV.

**Figure 1:**
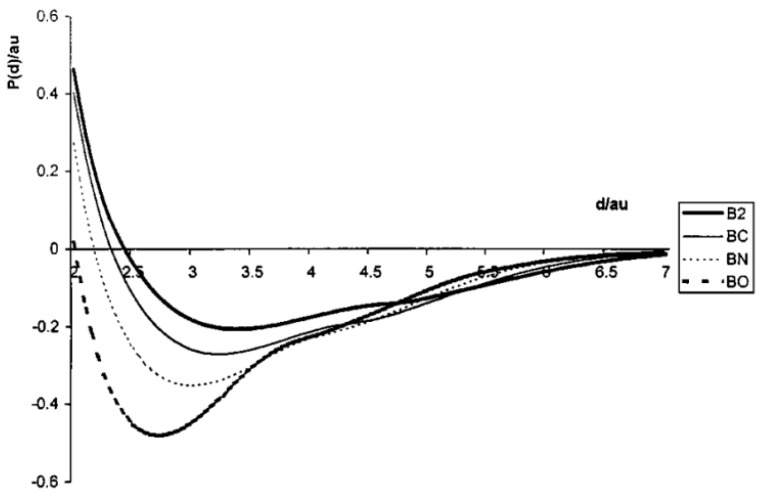
Quasi-classically calculated interatomic potentials for boron-containing diatomic molecules B_2_, BC, BN and BO versus inter-nuclear distances [42]. Copyright (2000), with permission from Elsevier.

Potential energy curves for the states of B_2_ were constructed by the complete-active-space SCF (Self Consistent Field) method at the multi-reference CI level [36]. According to other multi-reference CI study [40], its ground-state curve parameters—bond length, dissociation energy and relative vibration quantum are of 1.600–1.607 Å, 2.70–2.78 and 0.128–0.129 eV, respectively. For the B_2_ molecule ground-state interatomic potential energy, P curve P−d, where d is the inter-nuclear distance, constructed [41,42] within quasi-classical approach (Figure 1), the curve’s parameters are as follows: dissociation energy of 2.80 eV, equilibrium bond length of 1.78 Å and vibration quantum of 0.13 eV. Based on explicit expressions for intersite distances in boron nanotubes of regular geometry in terms of B−B bonds length and using this quasi-classical B−B pair potential, there were estimated some ground-state parameters of boron nanotubes [43,44,45].

The potential curves, transition energies, bond lengths and vibration frequencies of ground and some of low-lying excited electronic states of B_2_^+^ and B_2_ were obtained by using a CI approach [46]. The B_2_^+^ cation ground state, which was found to be ^2^Σ_g_^+^, shows a rather shallow potential curve with a bond length of 2.125 Å and vibration quantum of 0.052 eV, when compared with that of the ^3^Σ_g_^−^ state of B_2_ neutral: 1.592 Å and 0.131 eV. The first excited state of B_2_^+^, ^2^Π_u_, lies at 0.30 eV. As a result of loss of bonding electron, the ground-state dissociation energy for B_2_^+^ with calculated value of 1.94 eV is smaller than that of B_2_.

In Reference [47], the local nature of different types of boron–boron bonds from the topological analysis of ELF (Electron Localization Function) perspective was investigated in 23 boron-containing molecules.

Initially, Boustani demonstrated that stable structures of neutral bare boron clusters, B*_n_*, with *n* = 12, 16, 22, 32, 42 and 46, can easily be constructed with the help of the so-called Aufbau Principle suggested on the basis of HF SCF direct CI [48], as well as QC, DFT and LMTO (Linear Muffin Tin Orbital) [49,50] studies.

Based on different theoretical approaches, such as LDA (Local Density Approximation) and LSD (Local Spin Density) versions of DFT, HF, complete active space and scattered-wave SCF; correlated CI, QC, MO, PES (Potential Energy Surface) coupled-cluster, Born–Oppenheimer and full-potential LMTO versions of MD (Molecular Dynamics); and other methods, there are reported the key structural and binding parameters of boron clusters calculated or scaled from measured ones: B_3_ [37,51]; B_3_ and B_4_ in neutral and anionic forms [52]; B_4_ [53]; B_2_, B_3_ and B_4_ [54]; B_5_, B_5_^+^ and B_5_^−^ isomers [55]; B_5_^−^ [56]; B_6_ and B_6_^−^ [57]; B_7_ and B_7_^−^ [58]; B_2_^+^–B_8_^+^ [59]; B*_n_* with *n* = 2–8 in both the neutral and cationic states [60]; B*_n_* clusters with 4 ≤ *n* ≤ 8 [61]; 8- and 9-atom boron clusters [62]; small boron clusters with up to 10 atoms [63]; B_2–12_ and B_2–12_^+^ [64]; small neutral B*_n_* clusters with *n* = 2–12 [30]; icosahedral cluster B_12_ [65]; B_12_ [66,67]; B_2_^+^–B_13_^+^ [68] (see also Reference [69]); multi-charged clusters B*_n_* with *n* = 2–13 [70]; set of small-sized neutral B*_n_* and anionic B*_n_*^−^ boron clusters with *n* = 5–13 [31]; B_7_, B_10_ and B_13_ [71]; B_12_ and B_13_ for neutrals and cations [72]; B_12_^+^ and B_13_^+^ [73]; B_12_ and B_13_^+^ [74]; isomers of boron 13-clusters [75]; cationic, neutral and anionic charge states of B_13_ [76]; planar or quasi-planar structures of B_13_, B_13_^+^ and B_13_^−^ [77]; isomers of planar boron cluster B_13_ [78]; small cationic clusters B*_n_*^+^ with *n* = 2–14 [79]; B*_n_* with *n* = 2–14 [80,81]; B*_n_* clusters for *n* ≤ 14 [82]; B*_n_* with *n* = 5–14 [83]; boron clusters in the 10- to 15-atom size range [84]; B_16_^−^ and B_16_^2–^ [85]; B_19_^−^ [86]; neutral and anionic B_20_ isomers [87]; B_23_^+^ [88]; isomers of 24-atom boron cluster [89]; B_25_^−^ [90]; B_27_^−^ [91]; B*_n_* with *n* = 26–29 in both neutral and anionic states [32]; B_32_ [92]; B_36_ and B_36_^−^ [93,94]; B_41_^−^ and B_42_^−^ [95]; neutral B*_n_* clusters with *n* = 31–50 [33]; and boron cluster-families: spheres, double-rings and quasi-planars containing up to *n* = 122 atoms [96].

### 1.3. Applications

The unique behaviors of clusters of elemental boron have been identified: they react readily with metal surfaces; bond covalently to metal atoms; and cover surfaces with a boron-enriched hard, smooth and corrosion-resistant layer, which can be called nano-boron coating. Some parameters, especially feeding gas concentration, substrate temperature and input power, were optimized [97] to prepare high-pure boron (94%B) coating films by plasma-assisted CVD (Chemical Vapor Deposition). However, due to its thermal resilience, elemental boron is a difficult material to work with. To overcome this problem, Becker perfected a technique for generating clusters of elemental boron in plasma [98]. Unique tribological applications and scalability of boron-rich materials are likely to emerge from the combination of high mechanical strength, chemical stability, exceptional hardness and toughness, wear resistance, strong binding to substrates, low density and other promising physical–chemical properties [99].

The durability of boron coatings in sliding friction has been mentioned [100]. In the search ways to enhance the surface hardness of aluminum, the equilibrium structure, stability, elastic properties and formation dynamics of a boron-enriched surface were studied by using DFT [101]. Nano-indentation simulations suggested that the presence of boron nanostructures in the subsurface region significantly enhances the mechanical hardness of aluminum surfaces.

Boron is the only practical solid material with both volumetric and gravimetric energy densities substantially greater than those of hydrocarbons. That is why boron powder is attractive as a fuel or a supplement in propellants and explosives and potential source of secondary energy generation as well. One study [102] aimed to obtain the energy from elemental boron burning as solid fuel, which is synthesized from boron minerals. By experimental investigation [103] of the combustion characteristics of boron nanoparticles in the post-flame region, a two-stage combustion phenomenon was observed. The extended combustion model for single boron particles of sizes relevant for ramjet chambers was introduced [104] and validated [105]; it comprised a consistent formulation of the heat and mass transfer processes in the boron particles’ environment. A review on boron powders given in Reference [106] serves as the basis for research on the tendency of nanosized boron particles to group in an oscillating flow and its effect on the combustion process, flame characteristics and pollutants’ emission. Nanoboron can be considered as a superior rocket fuel because nano-particles have almost fluid-like properties. To optimize the reactive surface for combustion, nanoparticle size could be shrunk to clusters consisting of several atoms each. One review paper [107] encompassed the status and challenges in the synthesis process of boron nanoparticles, their dispersion and stability of in liquid hydrocarbon fuels, ignition and combustion characteristics of boron loaded liquid fuel, particle combustion and characterization of post-combustion products. The combustion characteristics of nanofluid fuels containing additions of boron and iron particles together were investigated in Reference [108]. Furthermore, mechanical milling was used to prepare a boron-based composite powder containing 5wt.% nanoiron to behave as a catalyst of boron oxidation [109]. The energy density of reactive metal fuel containing Ti, Al and B nanopowders was optimized [110] by varying the Ti:Al:B ratio.

As for information about oxidation of boron clusters, the cross-sections for ionic products formed in reactions of B_1–13_^+^ with oxygen were measured under single collision conditions [111] and three main reaction mechanisms found to be important: oxidative fragmentation, collision induced dissociation and boron atom abstraction. Cross-sections for oxidation reactions of CO_2_ with boron cluster ions B_1–14_^+^ were reported as a function of collision energy [112]. At least in some cases, oxidation causes structural rearrangement of the boron clusters. To rationally design and explore a future energy source based on the highly exothermic oxidation of boron, DFT was used to characterize small boron clusters with 0–3 oxygen atoms and, in total, up to 10 atoms [63].

Thin coatings made from the ^10^B isotopically enriched nanoboron providing the highest possible concentration of neutron capture centers can greatly simplify the problem of protection against thermal neutron irradiation [113,114,115], as well as neutron-fluence nanosensors [116,117]. Boron 2D metallic crystal is a prospective electromagnetic shielding nanomaterial as well [118], so nanoboron can combine neutron and electromagnetic shielding properties.

Reference [119] presented the concept that an elongated planar boron cluster can serve as a “tank tread” at the sub-nanometer scale, a novel propulsion system for potential nanomachines. Ferromagnetism in all-boron planar clusters, e.g., in B_34_, has been revealed theoretically [120]. They can be assembled to construct all-boron ferromagnetic monolayers, in which ferromagnetism–paramagnetism and semiconductor–metal transitions are expected to occur around 500 K, indicating their potential applications in nanoelectronic and spintronic devices at room temperature.

The recent review [121] on borophene potential applications discusses in detail its other utilizations, such as alkali metal ion and Li–S batteries, hydrogen storage, supercapacitors, sensor and catalytic in hydrogen evolution, oxygen reduction and evolution and CO_2_ electroreduction reaction.

The hierarchy of clusters relative stability/formation probability mainly (together with peculiarities characteristic of formation kinetics) is determined by the cluster-specific binding energy—its binding energy per atom. This work is focused on theoretical estimation of this key energy parameter for boron small planar clusters in frames of recently modified phenomenological diatomic molecular model.

## 2. Methodology

Specific binding energy or binding energy per chemical formula unit of the substance clustered form serves for important factor determining relative stabilities and, consequently, affects the relative concentrations of clusters with different numbers of formula units synthesized during a formation process (of course, the mentioned concentrations are influenced by the process kinetics as well). Here, we intend to calculate specific binding energies for boron small clusters in (quasi)planar structures, starting from the old diatomic model [122] of bounded multi-atomic structures.

The diatomic model is based on the saturation property of interatomic bonding. In its initial approximation, when binding energy is the sum of energies of pair interactions between only neighboring in the structure atoms, the microscopic theory of expansion allows for the quite correct estimation of the thermal expansion coefficient for crystals [123]. Despite its simplicity, the diatomic model has been successfully used to calculate some other anharmonic effects in solids as well [124].

As for the clusters binding energy, to the best our knowledge, there are no reports on its calculations within diatomic model, unless our previous estimates of small all-boron (quasi)planar clusters relative stabilities [125,126,127,128] and also their dipole moments [129,130]. These results have been summarized in the mini-reviews [131,132]. Furthermore, the problem was specially analyzed [133] for the three most abundant clusters, namely B_11_, B_12_ and B_13_, in different charge states, while taking into account ionization processes.

As is mentioned above, boron (quasi)planar clusters are of special interest, as they can form a borophene–monatomic boron layer with unique properties. The B–B bond length value obtained for boron finite planar clusters in quasi-classical approximation was used for an input parameter in quasi-classical calculations of electron energy band surfaces and DoS (Density-of-State) for a flat boron sheet with a perfect (i.e., without any type of holes) triangular network [134]. It is expected to have metallic properties. The Fermi curve of the boron flat sheet is found consist of 6 parts of 3 closed curves well-approximated by ellipses and then representing the quadric energy dispersion of the conduction electrons. The effective mass of electrons at the Fermi level is found to be too small compared with the free electron mass and highly anisotropic. The low effective mass of conduction electrons indicates their high mobility and, hence, high conductivity of the boron sheet.

Recently, we introduced [135] the most general formulation of the diatomic model allowing analytical calculation of the clusters binding energy. It is based on the following assumptions:

− Cluster binding energy is the sum of energies of pair interactions between nearest neighboring in its structure atoms;
− Assuming that relative deviations of bond lengths in the multi-atomic cluster structure from their values in corresponding diatomic molecules are small, pair interaction energies between neighboring atoms are approximated by their quadratic functions;
− Valence-electron-density-redistribution-related corrections to the bond energies in the cluster can be expressed through effective static charges localized on pairs of nearest neighboring atoms;
− Interatomic vibrations related corrections can be approximated by ground-state vibrational energies of corresponding diatomic molecules.

In the simplest but of practical interest special case, when all the bond lengths can be assumed to be almost equal each to other, a, cluster symmetry does not lead to any constrain (a relation to be satisfied by bond lengths), and most of other characteristics of valence bonding are equal each to other as well: $a_{0}$ is the bond length corresponding to diatomic molecule, $E_{0}$ is the diatomic molecule binding/dissociation energy, ω is the cyclic frequency of relative interatomic vibrations and M is the reduced mass of the pair of atoms with masses $\mu_{1}$ and $\mu_{2}$ constituting the bond:

(1) $$\frac{1}{M}=\frac{1}{\mu_{1}}+\frac{1}{\mu_{2}}$$

In case of identical atoms, we have the following:

(2) $$\mu_{1}=\mu_{2}\equiv \mu$$

and

(3) $$M=\frac{\mu }{2}$$

However, effective atomic charge numbers $Z_{i1}$ and $Z_{i2}$ characterizing electrostatic correction to the valence bonding energy remain different. The point is that the static charges localized on the pair of nearest-neighboring atoms differ for pairs placed at the center and periphery of the cluster, which is a finite structure of atoms. Here, the i index is as follows:

(4) i=1,2,3,…,k

where the numbers are different types of chemical bonds presented in the cluster, and k denotes their total number. If $N_{i}$ is the number of bonds of i-type, then we have the following:

(5) $$N=\overset{i=k}{\underset{i=1}{\sum }}N_{i}$$

which is the number of bonds in whole the cluster.

When we introduce the following parameter,

(6) $$Z=\overset{i=k}{\underset{i=1}{\sum }}N_{i}Z_{i1}Z_{i2}$$

equilibrium bonds length, a, and corresponding cluster binding energy, E (in Gauss units), are as follows:

(7) $$a=a_{0}+\frac{2e^{2}Z}{\mu \omega^{2}a_{0}^{2}N}$$

and

(8) $$E=\left(E_{0}-\frac{\hbar \omega }{2}\right)N-\frac{e^{2}Z}{a_{0}}+\frac{e^{4}Z^{2}}{\mu \omega^{2}a_{0}^{4}N}$$

respectively.

From the data available [39] for diboron molecule, $a_{0}$ ≈ 1.590 Å, $E_{0}$ ≈ 3.02 eV and ω ≈ 1051.3 cm^−1^ and boron atomic mass weighted for stable isotopes (^10^B and ^11^B) natural abundance μ ≈ 10.811 amu, we get the following formula for numerical calculations:

(9) $$E\left[\mathrm{eV}\right]\approx 2.995\;N-9.053\;Z+\frac{0.7273{\;Z}^{2}}{N}$$

The specific binding energy for cluster of n atoms is calculated as E/n.

To know parameter Z, one needs the estimates of effective atomic charges in the cluster. Below, they are found based on the assumption that the effective number of outer valence shell electrons localized on a given atomic site is proportional to its coordination number. Every neutral boron atom contains only 1 electron in the outer valence shell (2*p*-state). Then their total number in the all-boron cluster B*_n_* is given as follows:

(10) $$\nu =\{\begin{array}{l} n\;B_{n}^{0}\\ n-1\;B_{n}^{+}\\ n+1\;B_{n}^{-} \end{array}$$

Moreover, if $C_{j}$ denotes the coordination number of j-site, then we have the following:

(11) $$Z_{j}\approx 1-\frac{\nu C_{j}}{\sum_{l=1}^{l=n}C_{l}}$$

The numerical values of these effective charge numbers and a summary of them for parameter Z for the ground-state structures of boron planar clusters with n=2–15 atoms in three charge states are shown below in Table 1.

## 3. Results and Discussion

Here, we report the specific binding energy that we calculated for boron small planar clusters only in their ground-state structures. Ground-state structural isomers were chosen based on two criteria leading to maximal binding energy: (1) maximum number of bonds and (2) highest symmetry. We calculated clusters containing up to 15 boron atoms, because, for bigger species, 2D (quasi)planar structures are challenged by 3D ring-like structures (actually fragments of nanotubes), which are characteristic for boron clusters at n≥20.

**Figure 2:**
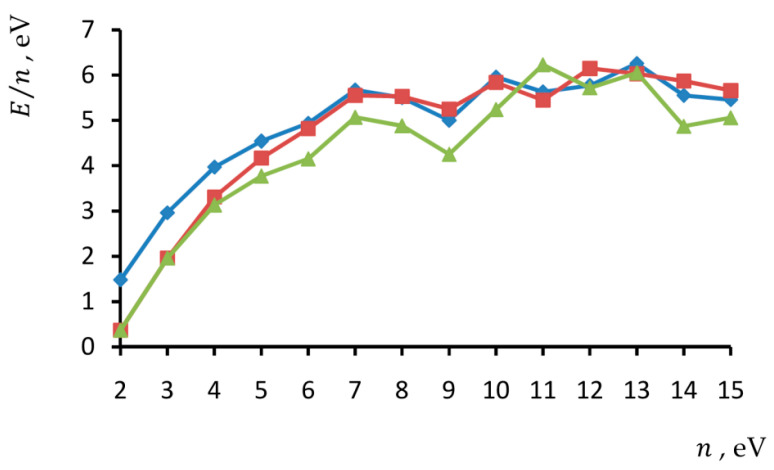
Specific binding energy of neutral (♦), positively (■) and negatively (▲) charged boron small planar clusters in dependence on number of atoms calculated in diatomic model.

Specific binding energies calculated in diatomic model for neutral B*_n_*^0^, cationic B*_n_*^+^ and anionic B*_n_*^−^ clusters with the number of atom n=2–15 are listed in Table 1 and presented in Figure 2.

**Figure 3:**
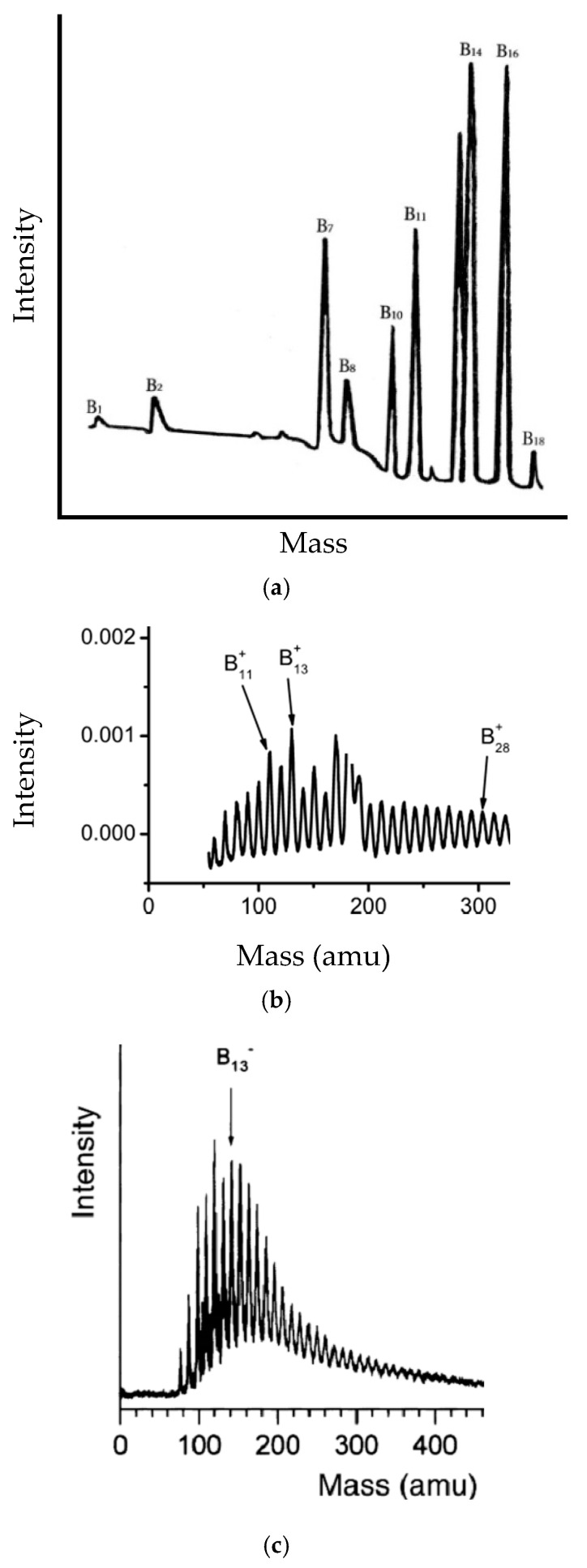
Experimentally recorded mass spectra of (**a**) neutral [130], Copyright (2015), with permission from Authors; (**b**) positively [29], Copyright (2010), with permission from KIT Scientific Publishing; and (**c**) negatively charged [28] boron clusters, Copyright (2003), with permission from Elsevier.

For comparison, available experimental data are presented in Figure 3. In Reference [130], clusters in boron vapor were generated by the thermal decomposition of electrode made of a boron-rich metal boride. Note that, in this way, initially clusters are formed in neutral state but then ionized by collisions with released in the chamber energetic electrons and accelerated toward the mass-spectrometer. Boron cluster cations were generated [29] by laser evaporation of target compact made of boron dust with gold added to increase its stability. Here, a disproportionately intense peak related to traces of gold is erased because it masks that of B^+^_18_, as masses of gold Au atom and boron 18-atom cluster are almost undistinguishable. As for the boron cluster anions, they were produced [28] by laser vaporization from homogeneous pure boron target rods.

**Figure 4:**
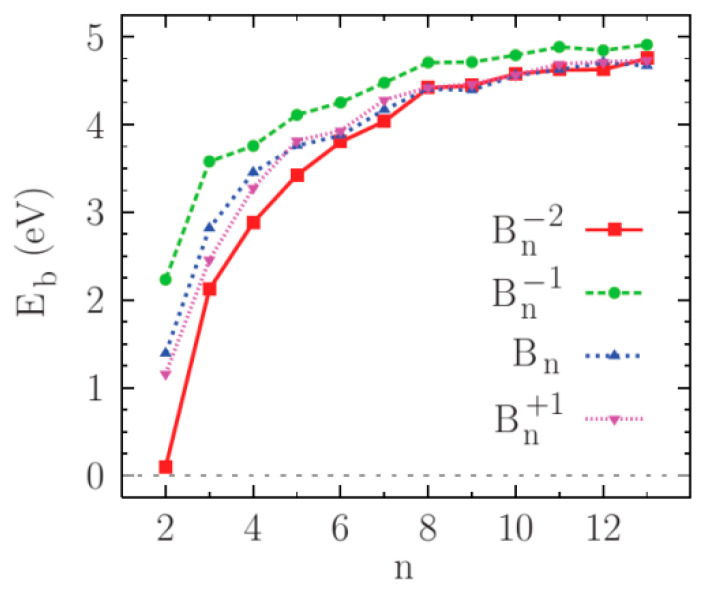
Binding energy per atom of neutral and ionized boron clusters as function of size [70], Copyright (2011), with permission from American Physical Society.

Again, for comparison, Figure 4 represents the theoretical specific binding energies of small boron clusters (in different, not only in planar, structures) depending on their size calculated by using QC methods [70]. One can note that such a typical curve looks similar to curves obtained in frames of diatomic model.

Thus, from the diatomic model, the theoretical equilibrium binding energies per B atom and B–B bond lengths are expected within ranges of 0.37–6.26 eV and 1.58–1.65 Å, respectively. For the most stabile neutral, positively and negatively charged species, B_13_^0^, B_12_^+^ and B_11_^−^ are predicted; their characteristics are 6.26, 6.15 and 6.23 eV and 1.59, 1.59 and 1.58 Å, respectively.

From experimental reports, the diboron molecule B_2_ dissociation energy is expected within the ranges of 2.58–3.06 [15] and 2.26–3.12 eV [34]. Theoretical values obtained by HF [37], MO [38], CI [40], quasi-classical [41,42] and PES [53] methods are 2.86, 2.71, 2.70–2.78, 2.80 and 2.70 eV, respectively. As diboron molecule contains two atoms, the corresponding specific binding energies equal to (2.58–3.06)/2 = 1.29–1.53 and (2.26–3.12)/2 = 1.13–1.56 for measured and 2.86/2 = 1.43, 2.71/2 ≈ 1.36, (2.70–2.78)/2 = 1.35–1.39, 2.80/2 = 1.40 and 2.70/2 = 1.35 eV for calculated dissociation energies. Calculations based on DFT [30] and QC [70] yielded 1.36 and 1.39 eV, respectively. The value of 1.48 eV obtained for cluster B_2_^0^ from the diatomic model falls in both experimental ranges and seems only slightly overestimated if compared with previous theoretical ones.

The ground-state dissociation energy of diboron cation B_2_^+^ calculated with the CI approach [46] is 1.94 eV, which corresponds to 1.94/2 = 0.97 eV for specific binding energy, significantly exceeding 0.37 eV yielded by diatomic model for B_2_^+^. The same is true for the QC specific binding energy for B_2_^+^: 1.16 eV [70]. This discrepancy should be related not only to the diatomic model itself but mainly phenomenological estimation of static atomic charges used. The point is that, when calculating static atomic charges in diatomic species, the equal dividing of a single elemental charge between constituent atoms is too crude of an approximation, leading to the overestimated Coulomb repulsive energy. SCF CI [69] and calibrated hybrid DFT [82] approaches’ B_2_^+^→B_1_^+^ + B_1_^0^ fragmentation energies are 1.47 and 1.96 eV, respectively. The corresponding specific binding energies are 1.47/2 ≈ 0.74 and 1.96/2 = 0.98 eV.

The QC specific binding energy of anionic cluster B_2_^−^ is 2.24 eV [70]. As for the cationic cluster, this value significantly exceeds 0.37 eV, which is suggested by the diatomic model. The reason should be the same as for cationic isomer.

For the B_3_ cluster, the HF scaled dissociation energy is 8.59 eV [37], while the PES study showed that atomization energy for B_3_ would be in the range of 8.21–8.36 eV [53]. As the molecule contains three atoms, the corresponding specific binding energies are equal to 8.59/3 ≈ 2.86 and (8.21–8.36)/3 ≈ 2.74–2.79 eV, respectively. Calculations based on DFT [30] and QC [70] approaches yielded 2.76 and 2.82 eV, respectively. The diatomic model’svalue of 2.96 eV for cluster B_3_^0^ again seems to be slightly overestimated if compared with other theoretical results.

The QC specific binding energy of B_3_^+^ and B_3_^−^ clusters is 2.46 and 3.58 eV, respectively [70], while the diatomic model gives 1.96 eV for both clusterions.

A SCF calculation [51] predicted that the energy of fragmentation of the B_3_ cluster to produce diatomic B_2_ and atomic B is 4.96 eV. From the diatomic model, one obtains 3 × 2.96 – 2 × 1.48 = 5.92 eV.

The SCF CI B_3_^+^→B_1_^+^ + B_2_^0^ and B_3_^+^→B_2_^+^ + B_1_^0^ fragmentation energies are 1.45 and 2.00 eV, respectively [68], while the B_3_^+^→B_1_^+^ + B_2_^0^ fragmentation energy calibrated in a hybrid DFT approach is 4.33 eV [82]. The diatomic model gives 3 × 1.96 − 2 × 0.37 = 5.14 and 3 × 1.96 − 2 × 1.48 = 2.92 eV for B_3_^+^→B_1_^+^ + B_2_^0^ and B_3_^+^→B_2_^+^ + B_1_^0^ reactions, respectively.

According to the study of the B_4_ cluster PES, its total atomization energy is expected in the range of 13.46–13.64 eV [53]. As this cluster consists of four atoms, the corresponding specific binding energy is (13.46–13.64)/4 ≈ 3.37–3.41 eV. The calculations based on DFT [30] and QC [70] approaches yielded 3.37 and 3.45 eV, respectively. The value of 3.97 eV obtained in the diatomic model for the neutral B_4_^0^ cluster is higher but comparable. However, the CI analysis of B_4_ tetramer in rhombus geometry significantly underestimated its specific binding energy: 2.42 eV [54].

The QC specific binding energies of B_4_^+^ and B_4_^−^ clusters are 3.28 and 3.76 eV, respectively [70]. The diatomic model gives quite close results of 3.31 and 3.13 eV for these cluster-ions.

SCF CI B_4_^+^→B_1_^+^ + B_3_^0^, B_4_^+^→B_3_^+^ + B_1_^0^ and B_4_^+^→B_2_^+^ + B_2_^0^ fragmentation energies are 1.75, 2.80 and 3.20 eV, respectively [68]. Moreover, B_4_^+^→B_1_^+^ + B_3_^0^ fragmentation energy calibrated in a hybrid DFT approach equals to 4.23 eV [82]. In the diatomic approach, their values are 4 × 3.31 − 3 × 2.96 = 4.36, 4 × 3.31 − 3 × 1.96 = 7.36 and 4 × 3.31 − 2 × 0.37 − 2 × 1.48 = 9.54, respectively.

The specific binding energies of the B_5_^0^ cluster calculated in DFT [30] and two versions of QC approach [31,70] are 3.67, 3.68 and 3.76 eV, respectively. Our result is 4.54 eV.

The QC specific binding energy of B_5_^+^ is 3.82 eV [70], while we obtained 4.17 eV.

The specific binding energies of the B_5_^−^ cluster calculated in two versions of the QC approach are 4.04 [31] and 4.11 eV [70], respectively, while the diatomic model yields the lower value of 3.77 eV.

The SCF CI B_5_^+^→B_1_^+^ + B_4_^0^ fragmentation energy is 2.00 eV [68]. By calibrating this energy in a hybrid DFT approach, it was found 4.95 eV [82] to be in almost perfect agreement with the diatomic model result of 5 × 4.17 − 4 × 3.97 = 4.97 eV.

By using DFT [30], QC [31,70] and diatomic model (this work) methods, we calculated the specific binding energy for the B_6_^0^ cluster as 3.79, 3.84, 3.88 and 4.94 eV, respectively. The QC approach [70] and diatomic model have yielded specific binding energy for B_6_^+^ cluster as 3.93 and 4.83 eV, respectively. According to two QC [31,70] and diatomic model calculations, the specific binding energy for B_6_^−^ cluster is 4.16, 4.25 and 4.15 eV, respectively.

The SCF CI B_6_^+^→B_5_^+^ + B_1_^0^ fragmentation energy is 2.20 eV [68], while, in a hybrid DFT approach, it is found to be 4.11 eV [82]. The diatomic value of 6 × 4.82 − 5 × 4.17 = 8.07 eV exceeds both of them.

DFT [30], QC [31,70], LMTO MD [71] and diatomic model specific binding energies for the B_7_^0^ cluster are 4.07, 4.11, 4.17, 5.24 and 5.67 eV, respectively. For the B_7_^+^ cluster, the QC [70] and diatomic-model specific binding energies are 4.28 and 5.55 eV, respectively. Moreover, the QC [31,70] and diatomic-model specific binding energies for B_7_^−^ cluster are 4.49, 4.48 and 5.07 eV, respectively.

The B_7_^+^→B_6_^+^ + B_1_^0^ fragmentation energy calibrated in a hybrid DFT approach is 6.05 eV [82], i.e., lower than that predicted by the diatomic model: 7 × 5.55 − 6 × 4.82 = 9.93 eV.

For the B_8_^0^ cluster, the DFT [30], QC [31,70] and diatomic-model specific binding energies are 4.31, 4.33, 4.41 and 5.50 eV, respectively. As for the B_8_^+^ cluster, its QC [70] and diatomic-model specific binding energies are 4.42 and 5.53 eV, respectively. Moreover, the B_8_^−^ cluster’s QC [31,70] and diatomic-model specific binding energies equal to 4.71, 4.71 and 4.88 eV, respectively.

The B_8_^+^→B_7_^+^ + B_1_^0^ fragmentation energy calibrated in a hybrid DFT approach is 5.04 eV [82], which is in good agreement with this work’s calculation: 8 × 5.53 − 7 × 5.55 = 5.39 eV.

The DFT [30], QC [70] and diatomic-model specific binding energies of B_9_^0^ cluster are 4.30, 4.39 and 5.00 eV, respectively. The QC [70] and diatomic-model specific binding energies of B_9_^+^ cluster are 4.46 and 5.25 eV, respectively. The QC [31,70] and diatomic-model specific binding energies of B_9_^−^ cluster are 4.55, 4.71 and 4.25 eV, respectively.

The B_9_^+^→B_8_^+^ + B_1_^0^ fragmentation energy calibrated in a hybrid DFT approach is 4.29 eV [82]. The diatomic model yields the fragmentation energy of 9 × 5.25 − 8 × 5.53 = 3.01 eV, which is surprisingly less than previous theoretical results.

The DFT [30], QC [70], LMTO MD [71] and diatomic-model specific binding energies of B_10_^0^ cluster are 4.45, 4.56, 5.78 and 5.96 eV, respectively. The QC [70] and diatomic-model specific binding energies of the B_10_^+^ cluster are 4.57 and 5.84 eV, respectively. The QC [70] and diatomic-model specific binding energies of the B_10_^−^ cluster are 4.79 and 5.24 eV, respectively.

As for the B_10_^+^→B_9_^+^ + B_1_^0^ fragmentation energy calibrated in a hybrid DFT approach, it equals 5.58 eV [82], while the diatomic model gives 10 × 5.84 − 9 × 5.25 = 11.15 eV.

The DFT [30], QC [70] and diatomic-model specific binding energies of the B_11_^0^ cluster are 4.52, 4.63 and 5.63 eV, respectively. QC [70] and diatomic-model specific binding energies of B_11_^+^ cluster are 4.69 and 5.45 eV, respectively. The QC [70] and diatomic-model specific binding energies of B_11_^−^ cluster are 4.89 and 6.23 eV, respectively.

The B_11_^+^→B_10_^+^ + B_1_^0^ fragmentation energy calibrated in a hybrid DFT approach is 5.18 eV [82]. In this case, the diatomic model value is significantly less: 11 × 5.45 − 10 × 5.84 = 1.55 eV.

In Reference [67], the Jahn–Teller distortion mechanism, which transforms the highlysymmetric icosahedral structure B_12_^0^ into a quasi-planar disc-like structure with binding energy per atom as 4.60 eV, was proposed. The same value was suggested based on DFT calculations: 4.60 eV [30]. The QC specific binding energy of B_12_^0^ is 4.71 eV [70], while our diatomic model gives 5.77 eV. The QC [70] and diatomic-model specific binding energies of the B_12_^+^ and B_12_^−^ charged cluster are 4.72 and 4.85, and 6.15 and 5.72 eV, respectively.

The B_12_^+^→B_11_^+^ + B_1_^0^ fragmentation energy calibrated in a hybrid DFT approach is 4.64 eV [82], while 12 × 6.15 − 11 × 5.45 = 13.85 eV is obtained based on diatomic model.

The QC [70], LMTO MD [71] and diatomic-model specific binding energies of the B_13_^0^ cluster are 4.67, 5.94 and 6.26 eV, respectively. The QC [70] and diatomic-model specific binding energies of the B_13_^+^ and B_13_^−^ charged cluster are 4.73 and 4.91, and 6.03 and 6.05 eV, respectively.

The B_13_^+^→B_12_^+^ + B_1_^0^ fragmentation energy calibrated in a hybrid DFT approach is 5.68 eV [82]. The diatomic model gives 13 × 6.03 − 12 × 6.15 = 4.59 eV.

Finally, the B_14_^+^→B_13_^+^ + B_1_^0^ fragmentation energy calibrated in a hybrid DFT approach is 3.96 eV [82], which is in satisfactory agreement with diatomic model value of 14 × 5.87 − 13 × 6.03 = 3.79 eV.

The above comparisons between the diatomic model and the previous results on specific binding and fragmentation energies of small boron clusters are summarized in Table 2 and Table 3, respectively. Here, the term “specific fragmentation energy” implies the fragmentation energy per doubled difference between B–B bonds’ numbers in the initial cluster (i.e., before fragmenting) and its fragments. For example, specific binding energies (in diatomic model), numbers of B atoms and B–B bonds of clusters B_4_^+^, B_2_^+^ and B_2_^0^ are 3.31, 0.37 and 1.48 eV; 4, 2 and 2; and 5, 1 and 1, respectively. Then, specific B_4_^+^→B_2_^+^ + B_2_^0^ fragmentation energy is (4 × 3.31 − 2 × 0.37 − 2 × 1.48)/(2 × (5 − 1 − 1)) = 1.59 eV.

Theoretical E/n−n curves obtained in the diatomic model in general features coincide with experimental ones. In particular, we can see maxima in the range B_11_–B_13_. At a number of atoms ≤8, neutral clusters are predicted to be more stable than their charged isomers. Moreover, at a number of ≤9, cations seem to be more stable than anions. However, at a higher number of atoms in boron planar clusters, there is no common trend of relative stability in the dependence of the charge state. As for the specific bind energy averaged by charge states, it saturates. Discrepancies in details seem to be related to the different kinetics of experimental generation processes of small boron clusters, as well as assumptions of the diatomic method used in theoretical calculations.

As for quantitative agreement in specific binding and fragmentation energies and their dependences on charge states and number of atoms in small boron clusters with previously reported (in the most part theoretical) studies, it also seems satisfactory. However, the diatomic model frequently overestimates these energy characteristics. This could be related to the energy phenomenological parameter—dissociation energy of diboron molecule B_2_—used in constructing the diatomic binding energy curve, as it is measured with too significant an error. Of course, such an almost systematic deviation from previous studies partially should be related to their assumptions on cluster structures and applied methods of calculation or measurement errors.

## 4. Conclusions

In summary, a previously developed diatomic-type model for calculating clusters’ specific (per atom) binding energy was applied to obtain this key parameter for boron small planar clusters’(B*_n_*, *n* = 2–15) ground-state isomers in different charge states. The main result of the conducted study was that, in any of the three considered (neutral, single-cationic and single-anionic) charge states, the formation of B_11_–B_13_ clusters is preferable. In general features, it is in good agreement with the available experimental data and previous theoretical reports.

As for quantitative agreement in specific binding and fragmentation energies, the diatomic model predicts the values in magnitude comparable with previous results, but it usually overestimates them. These deviations could be explained by different sets of assumptions made in theoretical calculations and/or too significant errors in experimental determination of boron clusters’ energy characteristics, one of which (B_2_ molecule dissociation energy), in particular, serves for key phenomenological parameter in diatomic model approach to calculating cluster specific binding energy.

Specific binding energies and B–B bond lengths of most stabile neutral, positively and negatively charged species are B_13_^0^—6.26 eV and 1.59 Å, B_12_^+^—6.15 eV and 1.59 Å and B_11_^−^—6.23 eV and 1.58 Å, respectively.

The success of the diatomic approach can serve for the basis for more detailed calculations of boron small clusters, including not only ground-state, but all the possible planar structural isomers, as well as competitive ring-like clusters. In this way, one can determine not only the specific binding energy but also other important characteristics, such as the cluster dipole moment, ionization potential and electron affinity, vibration, atomization, fragmentation energies, etc.

These results would be not only academic, but also practical interests, as boron (quasi)planar clusters serve for building blocks of borophene and other boron-based nanomaterials perspective for variety of technological applications, such as thin super-hard coatings, radiation shielding, solid fuel production, nanoelectronics, etc.

## Figures and Tables

**Table 1:** Specific binding energy of boron small planar clusters calculated in diatomic model.

Number of Atoms *n*	Structure	Number of Bonds *N*	Charge State	Charge Numbers in Dependence on Coordination Number					Parameter Z	Specific Binding Energy E/n, eV	Bonds Length, a, Å
				1	2	3	4	6			
2	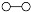	1	B_2_^0^	0					0	1.48	1.59
			B_2_^+^	$+\frac{1}{2}$					+0.2500	0.37	1.65
			B_2_^−^	$-\frac{1}{2}$					+0.2500	0.37	1.65
3	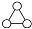	3	B_3_^0^		0				0	2.96	1.59
			B_3_^+^		$+\frac{1}{3}$				+0.3333	1.96	1.62
			B_3_^−^		$-\frac{1}{3}$				+0.3333	1.96	1.62
4	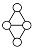	5	B_4_^0^		$+\frac{1}{5}$	$-\frac{1}{5}$			−0.1200	3.97	1.58
			B_4_^+^		$+\frac{2}{5}$	$+\frac{1}{10}$			+0.1700	3.31	1.60
			B_4_^−^		0	$-\frac{1}{2}$			+0.2500	3.13	1.60
5	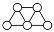	7	B_5_^0^		$+\frac{2}{7}$	$-\frac{1}{14}$	$-\frac{3}{7}$		−0.2194	4.54	1.58
			B_5_^+^		$+\frac{3}{7}$	$+\frac{1}{7}$	$-\frac{1}{7}$		−0.0204	4.17	1.59
			B_5_^−^		$+\frac{1}{7}$	$-\frac{2}{7}$	$-\frac{5}{7}$		+0.2041	3.77	1.60
6	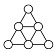	9	B_6_^0^		$+\frac{1}{3}$		$-\frac{1}{3}$		−0.3333	4.94	1.58
			B_6_^+^		$+\frac{4}{9}$		$-\frac{1}{9}$		−0.2593	4.82	1.58
			B_6_^−^		$+\frac{2}{9}$		$-\frac{5}{9}$		+0.1852	4.15	1.60
7	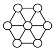	12	B_7_^0^			$+\frac{1}{8}$		$-\frac{3}{4}$	−0.4688	5.67	1.58
			B_7_^+^			$+\frac{1}{4}$		$-\frac{1}{2}$	−0.3750	5.55	1.58
			B_7_^−^			0		−1	0	5.07	1.59
8	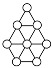	14	B_8_^0^		$+\frac{3}{7}$	$+\frac{1}{7}$	$-\frac{1}{7}$	$-\frac{5}{7}$	−0.2857	5.50	1.59
			B_8_^+^		$+\frac{1}{2}$	$+\frac{1}{4}$	0	$-\frac{1}{2}$	−0.3125	5.53	1.58
			B_8_^−^		$+\frac{5}{14}$	$+\frac{1}{28}$	$-\frac{2}{7}$	$-\frac{13}{14}$	+0.2589	4.88	1.60
9	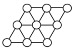	16	B_9_^0^		$+\frac{7}{16}$	$+\frac{5}{32}$	$-\frac{1}{8}$	$-\frac{11}{16}$	+0.2500	5.00	1.59
			B_9_^+^		$+\frac{1}{2}$	$+\frac{1}{4}$	0	$-\frac{1}{2}$	0	5.25	1.59
			B_9_^−^		$+\frac{3}{8}$	$+\frac{1}{16}$	$-\frac{1}{4}$	$-\frac{7}{8}$	+1.0000	4.25	1.61
10	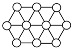	19	B_10_^0^			$+\frac{4}{19}$	$-\frac{1}{19}$	$-\frac{11}{19}$	−0.3850	5.96	1.59
			B_10_^+^			$+\frac{11}{38}$	$+\frac{1}{19}$	$-\frac{8}{19}$	−0.2465	5.84	1.59
			B_10_^−^			$+\frac{5}{38}$	$-\frac{3}{19}$	$-\frac{14}{19}$	+0.4127	5.24	1.60
11	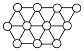	21	B_11_^0^		$+\frac{10}{21}$	$+\frac{9}{42}$	$-\frac{1}{21}$	$-\frac{4}{7}$	+0.0102	5.63	1.59
			B_11_^+^		$+\frac{11}{21}$	$+\frac{2}{7}$	$+\frac{1}{21}$	$-\frac{3}{7}$	+0.2381	5.45	1.59
			B_11_^−^		$+\frac{3}{7}$	$+\frac{1}{7}$	$-\frac{1}{7}$	$-\frac{5}{7}$	−0.7143	6.23	1.58
12	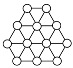	24	B_12_^0^			$+\frac{1}{4}$	0	$-\frac{1}{2}$	+0.1875	5.77	1.59
			B_12_^+^			$+\frac{5}{16}$	$+\frac{1}{12}$	$-\frac{3}{8}$	−0.3125	6.15	1.59
			B_12_^−^			$+\frac{3}{16}$	$-\frac{1}{12}$	$-\frac{5}{8}$	+0.2461	5.72	1.59
13	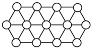	26	B_13_^0^			$+\frac{1}{4}$	0	$-\frac{1}{2}$	−0.5000	6.26	1.59
			B_13_^+^			$+\frac{4}{13}$	$+\frac{1}{13}$	$-\frac{5}{13}$	−0.1657	6.03	1.59
			B_13_^−^			$+\frac{5}{26}$	$-\frac{1}{13}$	$-\frac{8}{13}$	−0.2071	6.05	1.59
14	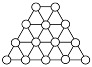	28	B_14_^0^		$+\frac{1}{2}$	$+\frac{1}{4}$	0	$-\frac{1}{2}$	+0.5625	5.55	1.60
			B_14_^+^		$+\frac{15}{28}$	$+\frac{17}{56}$	$+\frac{1}{14}$	$-\frac{11}{28}$	+0.0590	5.87	1.59
			B_14_^−^		$+\frac{13}{28}$	$+\frac{11}{56}$	$-\frac{1}{14}$	$-\frac{17}{28}$	+1.6151	4.87	1.61
15	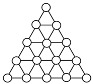	30	B_15_^0^		$+\frac{1}{2}$		0	$-\frac{1}{2}$	+0.7500	5.46	1.60
			B_15_^+^		$+\frac{8}{15}$		$+\frac{1}{15}$	$-\frac{2}{5}$	+0.4133	5.66	1.59
			B_15_^−^		$+\frac{7}{15}$		$-\frac{1}{15}$	$-\frac{3}{5}$	+1.4133	5.06	1.60

**Table 2:** Comparison of the literature data on specific binding energies (in eV) of boron small clusters with values calculated in diatomic model.

Cluster	Literature Data	Diatomic Model
B_2_^0^	1.29–1.53 [15] 1.36 [30] 1.13–1.56 [34] 1.43 [37] 1.36 [38] 1.35–1.39 [40] 1.40 [41,42] 1.35 [53] 1.39 [70]	1.48
B_2_^+^	0.97 [46] 0.74 [69] 1.16 [70] 0.98 [82]	0.37
B_2_^−^	2.24 [70]	0.37
B_3_	2.76 [30] 2.86 [37] 2.74–2.79 [53] 2.82 [70]	2.96
B_3_^+^	2.46 [70]	1.96
B_3_^−^	3.58 [70]	1.96
B_4_	3.37 [30] 3.37–3.41 [53] 2.42 [54] 3.45 [70]	3.97
B_4_^+^	3.28 [70]	3.31
B_4_^−^	3.76 [70]	3.13
B_5_^0^	3.67 [30] 3.68 [31] 3.76 [70]	4.54
B_5_^+^	3.82 [70]	4.17
B_5_^−^	4.04 [31] 4.11 [70]	3.77
B_6_^0^	3.79 [30] 3.84 [31] 3.88 [70]	4.94
B_6_^+^	3.93 [70]	4.83
B_6_^−^	4.16 [31] 4.25 [70]	4.15
B_7_^0^	4.07 [30] 4.11 [31] 4.17 [70] 5.24 [71]	5.67
B_7_^+^	4.28 [70]	5.55
B_7_^−^	4.49 [31] 4.48 [70]	5.07
B_8_^0^	4.31 [30] 4.33 [31] 4.41 [70]	5.50
B_8_^+^	4.42 [70]	5.53
B_8_^−^	4.71 [31] 4.71 [70]	4.88
B_9_^0^	4.30 [30] 4.39 [70]	5.00
B_9_^+^	4.46 [70]	5.25
B_9_^−^	4.55 [31] 4.71 [70]	4.25
B_10_^0^	4.45 [30] 4.56 [70] 5.78 [71]	5.96
B_10_^+^	4.57 [70]	5.84
B_10_^−^	4.79 [70]	5.24
B_11_^0^	4.52 [30] 4.63 [70]	5.63
B_11_^+^	4.69 [70]	5.45
B_11_^−^	4.89 [70]	6.23
B_12_^0^	4.60 [30] 4.60 [67] 4.71 [70]	5.77
B_12_^+^	4.72 [70]	6.15
B_12_^−^	4.85 [70]	5.72
B_13_^0^	4.67 [70] 5.94 [71]	6.26
B_13_^+^	4.73 [70]	6.03
B_13_^−^	4.91 [70]	6.05

**Table 3:** Comparison of the literature data on specific fragmentation energies (in eV) of boron small clusters with values calculated in diatomic model.

Chanel	Literature Data	Diatomic Model
B_3_^0^→B_2_^0^ + B_1_^0^	1.24 [51]	1.48
B_3_^+^→B_2_^0^ + B_1_^+^	0.36 [68] 1.08 [82]	1.29
B_3_^+^→B_2_^+^ + B_1_^0^	0.50 [68]	0.73
B_4_^+^→B_3_^0^ + B_1_^+^	0.44 [68] 1.06 [82]	1.09
B_4_^+^→B_3_^+^ + B_1_^0^	0.70 [68]	1.84
B_4_^+^→B_2_^0^ + B_2_^+^	0.80 [68]	2.39
B_5_^+^→B_4_^0^ + B_1_^+^	0.50 [68] 1.24 [82]	1.24
B_6_^+^→B_5_^+^ + B_1_^0^	0.55 [68] 1.03 [82]	2.02
B_7_^+^→B_6_^+^ + B_1_^0^	1.01 [82]	1.66
B_8_^+^→B_7_^+^ + B_1_^0^	1.26 [82]	1.35
B_9_^+^→B_8_^+^ + B_1_^0^	1.07 [82]	0.75
B_10_^+^→B_9_^+^ + B_1_^0^	0.93 [82]	1.86
B_11_^+^→B_10_^+^ + B_1_^0^	1.30 [82]	0.39
B_12_^+^→B_11_^+^ + B_1_^0^	0.77 [82]	2.31
B_13_^+^→B_12_^+^ + B_1_^0^	1.42 [82]	1.15
B_14_^+^→B_13_^+^ + B_1_^0^	0.99 [82]	0.95

## Data Availability

Data available via personal communication with proper reasons.

## Sample Availability

Samples of the compounds are not available from the authors.
